# Pretest PSA and Restaging PSMA PET/CT Predict Survival in Biochemically Recurrent Prostate Cancer

**DOI:** 10.3390/biomedicines11092333

**Published:** 2023-08-22

**Authors:** Rie von Eyben, Manuela Andrea Hoffmann, Cigdem Soydal, Irene Virgolini, Murat Tuncel, Mathieu Gauthé, Daniel S. Kapp, Finn Edler von Eyben

**Affiliations:** 1 Cytel Incorporated, Waltam, MA 02452, USA; 2 Institute for Preventive Medicine of German Armed Forces, 56626 Andernach, Germany; 3 Department of Nuclear Medicine, University Medical Center, Johannes Gutenberg University in Mainz, 55131 Mainz, Germany; 4 Department of Nuclear Medicine, University of Ankara, 0600 Ankara, Turkey; csoydal@yahoo.com; 5 Department of Nuclear Medicine, University Hospital in Innsbruck, 6020 Innsbruck, Austria; irene.virgolini@i-med.ac.at; 6 Department of Nuclear Medicine, Hacettepe University, Ankara 06230, Turkey; 7 Department of Nuclear Medicine, Incept, Institute Holland, 38100 Grenoble, France; mathieugauthe@yahoo.fr; 8 Department of Radiation Oncology, Stanford University School of Medicine, Stanford, CA 94305, USA; 9 Center of Tobacco Control Research, DK-5230 Odense, Denmark

**Keywords:** Cox regression analysis of survival, prostate specific antigen (PSA) relapse, prostate specific membrane antigen (PSMA) PET/CT, restaging

## Abstract

Background: A biochemical recurrence (BCR) risk model was created based on pretest prostate specific antigen (PSA) and groupings by restaging prostate specific membrane antigen (PSMA) PET/CT. Methods: A cohort of 1216 BCR patients were analyzed for overall survival (OS) according to the PSA threshold and restaging PSMA PET/CT. A Cox regression analysis of OS was carried out to detect significant clinical characteristics. Results: In the cohort, 271 patients had a pretest PSA of <0.5 ng/mL and 945 patients had higher PSA values. The restaging PSMA PET/CT was positive for 834 patients and negative for 369. Of 1203 patients, 133 (11%) died, including 19 of the 369 (5%) patients without positive sites on the restaging PSMA PET/CT, 82 of the 711 (12%) with 1–5 positive sites, and 32 of the 123 (26%) with >5 positive sites. In the Cox regression analysis, four variables significantly predicted OS: treatment center, International Society of Urologic Pathology (ISUP) grade, pretest PSA threshold, and the grouping of positive sites on the restaging PSMA PET/CT. Conclusions: The pretest PSA and PSMA PET/CT were important for the OS of the BCR patients. The findings argue for the new BCR risk model and serve as framework for ongoing trials.

## 1. Introduction

Prostate cancer (PC) is a frequent male cancer with a high mortality [1]. Patients with localized PC are treated with radical prostatectomy (RP) or radiation therapy (RT), or with RP and RT combined with androgen deprivation therapy (ADT). After initial treatment, up to half of patients develop a recurrence. First, the recurrent patients have rising prostate specific antigen (PSA) values, despite conventional imaging not showing PC lesions. The phase is called biochemical recurrence (BCR, prostate specific antigen (PSA) relapse, or failure). BCR increases the risk of dying of PC [2]. 

Danish, German, and international guidelines recommend that BCR patients are restaged with prostate specific membrane antigen (PSMA) PET/CT [3], and that nearly all BCR patients undergo salvage treatment while their PSA is <0.5 ng/mL [4]. A multicenter study of 1216 BCR patients showed that the PSA threshold had a significant impact on their overall survival (OS) [5]. The European Association of Urology (EAU) published a BCR risk classification [6], and later, the multicenter study made a new BCR risk model that was based on the pretest PSA threshold and groups by number of positive sites on a restaging PSMA PET/CT [7]. 

Using a Cox regression analysis, our multicenter study of BCR patients aims to discover whether pretest PSA, restaging PSMA PET/CT, and other clinical characteristics are significantly associated with OS. 

## 2. Materials and Methods

### 2.1. Patients

BCR patients were recruited between Dec 2014 and April 2020 at five centers across two continents [5,7]. The University in Ankara, Turkey, reported 94 patients; the Hacettepe University, Ankara, Turkey, reported 102; the University Hospital in Innsbruck, Austria, reported 500; the Johannes Gutenberg University in Mainz, Germany, reported 225; and Sorbonne University, Paris, France, reported 294 patients. 

Initially, the patients had undergone RP or RT, and all had BCR. The patients were >18 years at diagnosis, had histologically proven PC, were evaluated in an early phase of BCR, and were followed for a median of >2 years after BCR. All the patients provided informed consent to the study. On 26 October 2021, the Ankara University Medical School, Ankara, Turkey, approved the study (reference number 19-607-21).

### 2.2. PSA and PSMA PET/CT

PSA was measured with ultrasensitive methods [5]. Restaging PSMA PET/CT scans were conducted as [^68^Ga]Ga-PSMA-11, according to international guidelines [8]. Four centers used PSMA PET/CT for all BCR patients, whereas the Paris center used PSMA PET/CT only for patients with a negative choline PET/CT.

### 2.3. Definitions

Patients were defined to have BCR if two PSA measurements were obtained with at least a one-week interval and showed rising values. RP patients were defined to have BCR if their PSA declined after RP to unmeasurable levels and later increased. After RT, patients were defined to have BCR if their PSA declined after RT to a PSA nadir and later increased. The time to BCR was defined as the time from the initial treatment to BCR. 

In the EAU BCR risk classification [6], the low-risk group for RP patients was defined as patients with an International Society of Urologic Pathology (ISUP) [9] grades of 1–3 and a PSA doubling time (PSADT) of >12 months. The high risk RP patients were defined as those with ISUP grades of 4–5 and a PSADT of <12 months. The low-risk RT patients were defined as those with biopsy ISUP grades of 1–3 and a time to BCR of >18 months. The high-risk RT patients were defined as those with biopsy ISUP grades 4–5 and a time to BCR < 18 months. The high-risk RT group of patients was defined as patients with a biopsy ISUP grade of 4–5 or a time interval to BCR of ≤18 months. 

OS was defined as the time from the restaging PSMA PET/CT to death of any cause, or as the time alive to end of the follow-up.

### 2.4. Statistical Analysis

We did not impute missing data. We evaluated the characteristics of the patients below or above the PSA threshold of 0.5 ng/mL. A Cox proportional hazard regression analysis of the OS evaluated the potentially predictive characteristics and was carried out with backward elimination of the insignificant variables. For the significant variables, hazard ratios and their 95% confidence intervals (CI) were calculated. We truncated Kaplan–Meier plots of the OS at 5 years after BCR. A combined p value was based on the p values on a topic in several studies and calculated in STATA using the method of Tobias [10].

All the tests were two-sided and a *p* value of <0.05 indicated statistical significance. The statistical analyses were conducted in SAS (SAS institute Inc., Cary, NC, USA) or in STATA version 17.0 (Stata Corp., College Station, TX, USA).

## 3. Results

### 3.1. Patient Characteristics

The clinical characteristics of the patients are shown in Table 1. Overall, the PSA immediately before or at restaging PSMA PET/CT (pretest PSA) did not differ significantly between the Ankara University patients and the patients from the Hacettepe, Mainz, Innsbruck, and Paris centers (*p* = 0.56, *t*-test), but only 10% of the Ankara University patients had a pretest PSA of <0.5 ng/mL, in contrast to 25% for the patients at the four other centers (*p* = 0.002, chi^2^ test). 

Initially, the patients were treated with RP (with or without ADT) or RT. At restaging PSMA PET/CT, RP and RT patients did not differ significantly in terms of the number of positive sites on the restaging PSMA PET/CT (*p* = 0.08, chi^2^ test, Table 2). The five cohorts of patients differed markedly in their OS (*p* < 0.00005, log-rank test). 

Age at diagnosis, T stage, and retest PSA were significantly associated with the grouping by number of positive sites on the restaging PSMA PET/CT, as shown in Table 2. 

The patients in all ISUP grades differed in their OS (*p* < 0.00005, log-rank test). However, lumping the ISUP grades into two categories (grade 1–3 vs. 4–6) was strongly associated with groupings of positive sites on the restaging PSMA PET/CT (*p* < 0.0005, chi^2^ test). 

### 3.2. Pretest PSA and Restaging PSMA PET/CT

**Figure 1:**
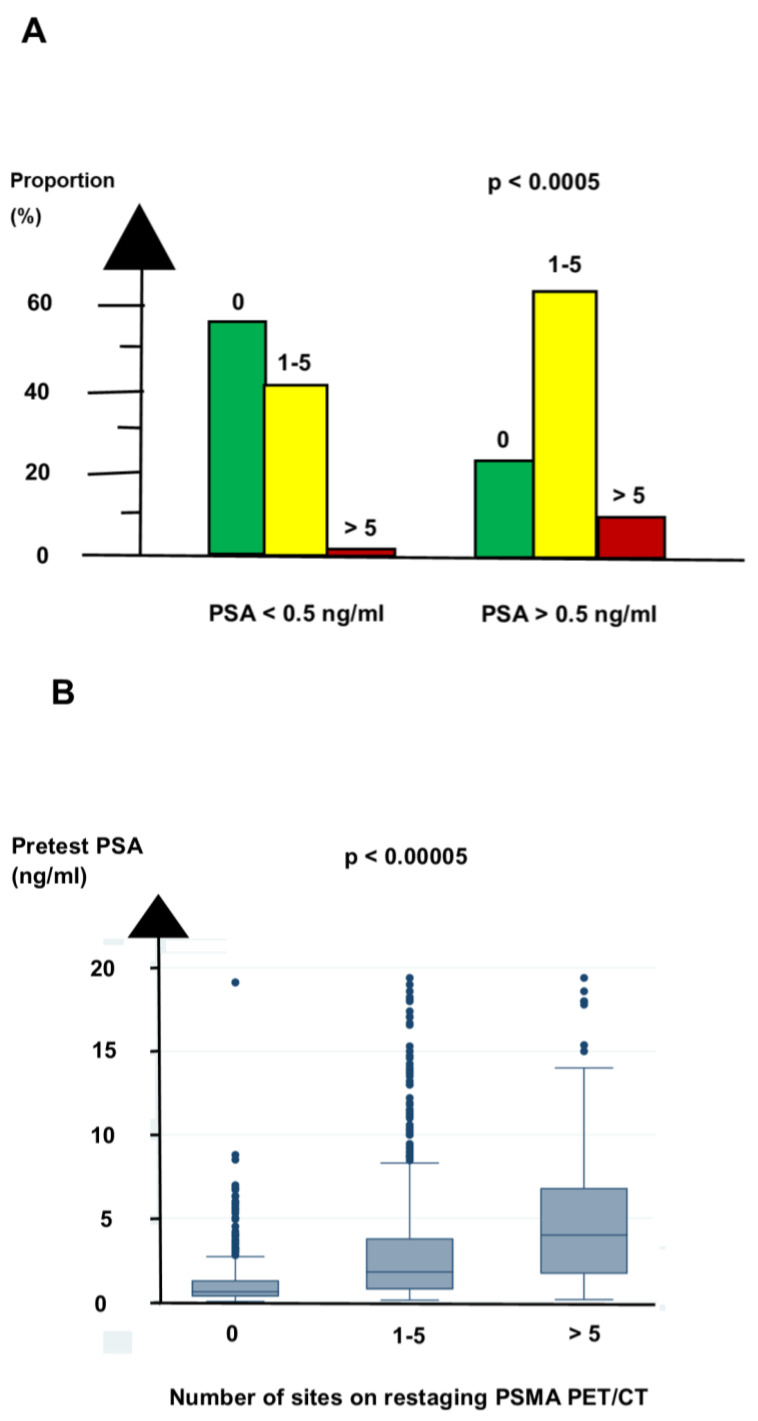
The association between pretest PSA and restaging PSMA PET/CT. (**A**) The grouping by number of sites on restaging PSMA differed between the patients with a PSA below and above the PSA threshold of 0.5 ng/mL. (**B**) The level of pretest PSA differed between the groupings by number of sites on restaging PSMA PET/CT. The y axis on (**B**) is truncated at pretest PSA 20 ng/mL. Abbreviation: PSA = prostate specific antigen; PSMA = prostate specific membrane antigen.

A pretest PSA of <0.5 ng/mL was associated with a negative PSMA PET/CT for most patients and with polysites for few patients, as shown in Table 3 and Figure 1A. A pretest PSA of ≥0.5 ng/mL was associated with polysites on the PSMA PET/CT for more patients than a pretest PSA < 0.5 ng/mL.

Patients with polyrecurrent PC had a median pretest PSA that was nearly ten times the median pretest PSA for patients with a negative PSMA PET/CT, as shown in Figure 1B. 

### 3.3. Treatment

The first salvage treatment of BCR was known for <20% of the patients. Patients with a negative PSMA PET/CT were mainly given salvage RT with or without ADT, and the treatment was also given to some patients with 1–5 and >5 positive sites. Patients with regional or distant sites on PSMA PET/CT were mainly given systemic treatment. 

### 3.4. Pretest PSA, Restaging PSMA PET/CT, and Mortality

The patients were followed until autumn 2021. Overall mortality was 11%. This mortality rose from 19 of the 369 (5%) patients without positive sites on their restaging PSMA PET/CT, from 82 of the 711 (12%) patients with 1–5 positive sites, and 32 of the 123 (26%) patients with >5 positive sites. 

Patients with a pretest PSA of <0.5 ng/mL had an excellent OS. For these patients, OS was influenced little by the groupings by number of positive sites on restaging PSMA PET/CT (*p* = 0.056, log-rank test). 

### 3.5. Pretest PSA, Restaging PSMA PET/CT, EAU BCR Risk Score, and Overall Survival 

**Figure 2:**
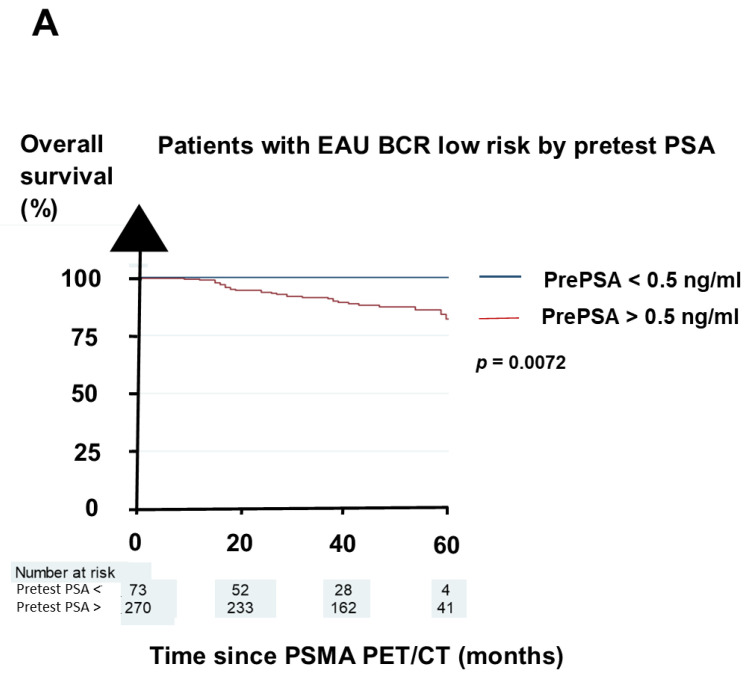

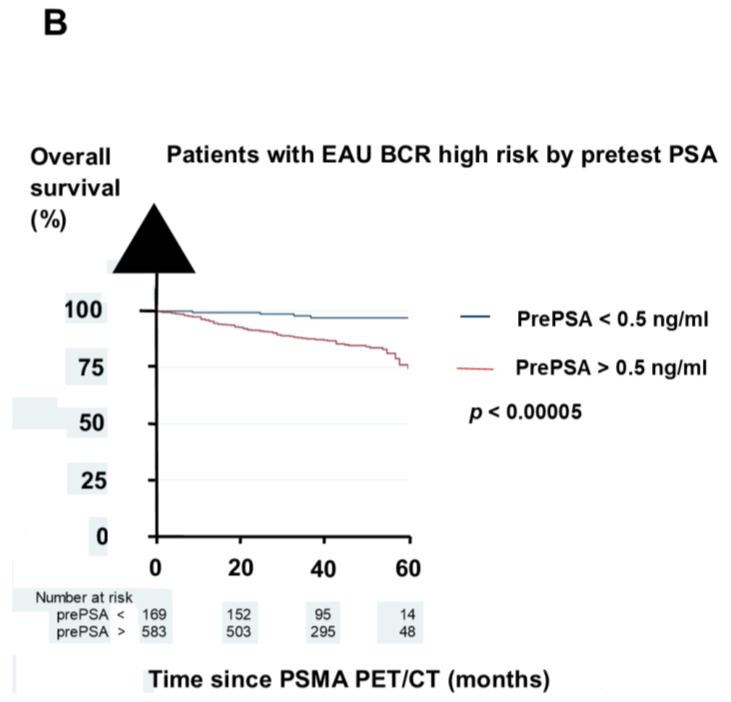
The pretest PSA threshold separated the two EAU BCR risk groups into two groups that differed markedly in overall survival. (**A**) EAU BCR low-risk patients. (**B**) EAU BCR high-risk patients. Abbreviation: BCR = biochemical recurrence; EAU = European Association of Urology; and PrePSA = pretest PSA.

**Figure 3:**
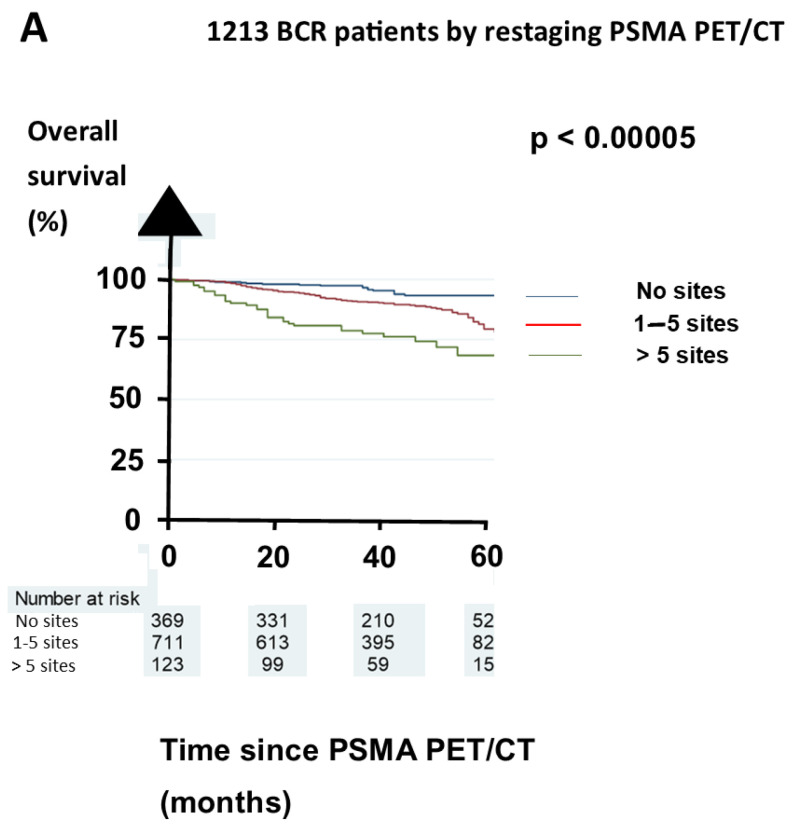

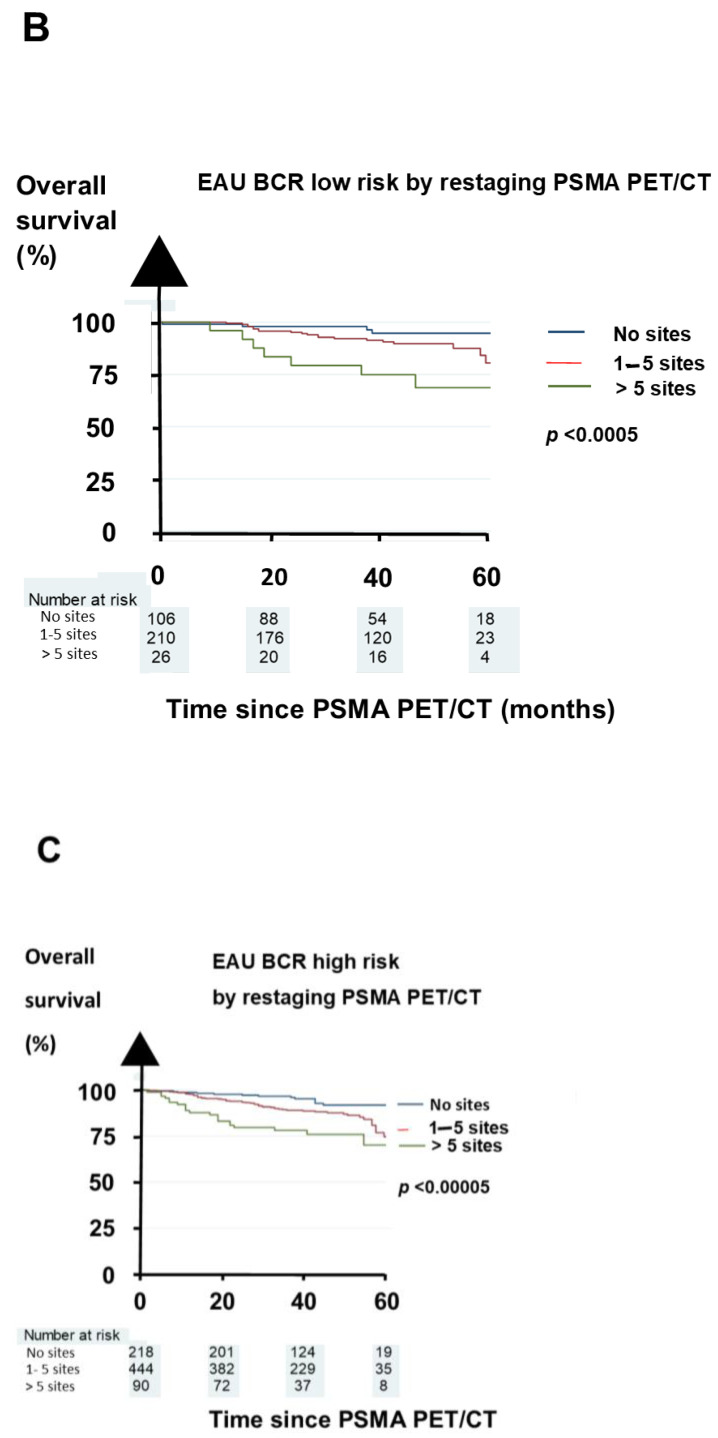
The overall survival for the three groups by numbers of positive sites on restaging PSMA PET/CT. (**A**) The total cohort of patients. (**B**) The EAU BCR low-risk group. (**C**) The EAU BCR high-risk group. Abbreviation: PSMA = prostate membrane specific antigen. Patients with a negative PSMA PET/CT are shown with a black survival line, patients with 1–5 positive sites are shown with a red line, and patients with more than 5 positive sites are shown with another black line.

Overall, 343 (28%) patients had EAU BCR low risk, 752 (62%) had EAU BCR high risk, and 121 (10%) were not classifiable for the EAU BCR risk. The OS differed significantly between the subgroups in terms of the pretest PSA threshold of 0.5 ng/mL [5]. Regarding the two EAU BCR risk groups, the two subgroups by the PSA threshold of 0.5 ng/mL had a grossly similar OS, as shown in Figure 2A,B. The OS was also similar for the three groups in terms of the number of positive sites on the restaging PSMA PET/CT for all the patients (Figure 3A), the EAU BCR low-risk patients (Figure 3B), and the EAU BCR high-risk patients (Figure 3C). 

For the EAU BCR high-risk patients, those with a pretest PSA of <0.5 ng/mL had a >95% 5-year OS. For the EAU BCR low-risk patients, those with polysites on their PSMA PET/CT had 5-year OS of <80%. 

### 3.6. Cox Regression Analysis of Overall Survival

In the Cox regression analysis of the OS, most clinical characteristics were statistically insignificant, as shown in Table 4. After the backward elimination of the insignificant variables, the final prognostic model included four variables: treatment center, ISUP grade, pretest PSA threshold, and grouping of positive sites on restaging PSMA PET/CT (Table 5). The prognostic model had C statistics of 0.75. 

## 4. Discussion

Our study adds important information for the association between pretest PSA, restaging PSMA PET/CT, and OS. The review has the most extensive analyses so far of the factors for OS in patients after a restaging PSMA PET/CT. The dichotomy in terms of the PSA threshold of 0.5 ng/mL had greater impact on the OS than the dichotomy between the PSMA PET/CT-negative patients and those with oligorecurrent PC, and the dichotomy between oligorecurrent PC and polyrecurrent PC.

Our study differs from studies that have evaluated whether salvage radiation therapy should only target the prostate bed or target both the prostate bed and the pelvic lymph nodes. Our study also differs from studies that have evaluated whether radiation therapy should be combined with ADT. Our study also differs from studies that used change of planned treatment after a restaging PSMA PET/CT and delay of ADT as end points. It remains to be proven whether such changes prolong OS. OS, our alternative end point, is clinically relevant.

In our study, centers contributed significantly to the heterogeneity in the OS after BCR. In part, the heterogeneity was due to the tumor burden at the BCR. Paris patients had a relatively small tumor burden, and patients at the Ankara University Center had a relatively large tumor burden.

Especially the ISUP grades 3 and 4, differed in their impact on the OS. Our Cox regression analysis showed that the individual ISUP gradings were more strongly associated with OS than lumping ISUP into two groups of patients: those with ISUP grades of 1–3 and those with ISUP grades of 4–5.

A pretest PSA threshold of 0.5 ng/mL was more strongly associated with OS than the individual PSA values. Like our study, a recent study pointed to the use of an ultrasensitive PSA assay to monitor increases at very low PSA values before BCR [11]. The EAU PC guidelines of 2021 defined BCR as a rising PSA, and did not include a low PSA threshold.

Two previous studies also reported that a BCR patient with a pretest PSA of <0.5 ng/mL had an excellent OS [12,13]. In a third study, 109 of 137 (80%) patients had a pre-treatment PSA below the PSA threshold [14]. Of the 109 patients, 74 (68%) had a negative PSMA PET/CT or positive sites in the prostate bed, and 35 (32%) patients had regional or distant positive sites. The combined *p* value for a PSA threshold of 0.2–0.5 ng/mL at BCR based on in the studies was <4 × 10^−12^.

Of our patients, like patients at other centers [15], some with RT BCR started SRT with a pretreatment PSA lower than 2+ ng/mL above the post-RT PSA nadir. That is the Phoenix criterion to indicate BCR and to trigger SRT [16].

In our study, three groups by lumping sites on the PSMA PET/CTs were more strongly associated with OS than the individual number of positive sites. By lumping the PET/CT sites, the three groups related more strongly to the OS than three groups by the regional location of the positive sites. Our study indicated that a PET/CT positive site in the prostate bed was as important for the OS as a positive site elsewhere.

Our three groups were also more strongly associated with OS than the regional location of the positive sites. Pretest PSA was significantly associated with restaging PSMA PET/CT, shown in Figure 1. But both clinical variables significantly predicted OS in Cox regression analysis.

Like in our study, another study on BCR reported that 68% of patients with a negative restaging PSMA PET/CT lived three years free of BCR compared to 32% of patients with a positive PSMA PET/CT [17]. A third study showed that the number of positive sites on the restaging PSMA PET/CT gave a marked difference in the 1.5-year survival free of BCR: 77% for one lesion vs. 30% for three to five lesions (*p* = 0.022, log-rank test) [18]. However, a fourth study showed that 10 of 35 (28%) PET-negative patients had imaging progression, whereas 21 of 65 (31%) PET-positive patients had imaging progression [19]. In the study, the EAU BCR low-risk patients were less often treated for relapse than the EAU BCR high-risk patients.

Our study is the first to analyze whether the components in the EAU BCR risk classification might have an individual significant prognostic value. The only significant prognostic factor was the ISUP grading. Similarly, the EAU BCR risk classification did not have an impact on the OS known from the groupings by pretest PSA threshold and restaging PSMA PET/CT, as shown in Figure 2 and Figure 3.

The restaging PSMA PET/CTs showed heterogeneity in the EAU BCR risk groups, confirming the findings of three other studies [20,21,22]. A fourth study reported that 427 of 2140 (32%) Spanish RP patients had BCR [23], without the mortality being markedly different between the EAU BCR low- and high-risk patients.

Salvage treatment contributes to the BCR heterogeneity in OS. In general, oligorecurrent BCR patients diagnosed with ^68^Ga-PSMA PET/CT are treated with ADT, but some patients are given metastases-directed RT (MDT) [24].

Ongoing BCR trials study salvage treatment based on restaging PSMA PET/CTs. The Peryton trial evaluates patients with negative PSMA PET/CTs and compares hypo-fractionated and conventionally fractionated RT [25]. The ADOPT trial studies oligorecurrent patients and evaluates whether ADT adds to MDT [26].

New multicenter trials may improve outcomes by a common protocol of the strict monitoring of PSA using ultrasensitive PSA assays during follow-up, and by a common protocol for salvage treatment. The principles may reduce or eliminate the prognostic impact of the treatment center in our risk model. Outcomes may especially be improved by increasing the proportion of BCR patients with a pretest PSA below the PSA threshold of 0.5 ng/mL before salvage treatment.

Our study has strengths and limitations. A strength is the concordance between our findings and those reported in the literature. The similarity gives our findings external validity. The new model uses both PSA and PSMA PET/CT, which are already recommended in recent international guidelines. Its limitations are the retrospective study design and the few deaths during the short follow-up. Another limitation is the risk of false-positive or false-negative restaging PSMA PET/CTs. At present, some countries have limited access to or limited use of PSMA PET/CTs.

In conclusion, pretest PSA threshold and groupings by restaging PSMA PET/CT show BCR heterogeneity in terms of OS. The guidelines recommend the two tests [3,4] and they are combined in a new BCR risk model [6] that serves as framework for ongoing trials.

## Figures and Tables

**Table 1:** Clinical characteristics and pretest PSA.

Characteristics		Results			*p* Value
		Total cohort	Pretest SSA < 0.5 ng/mL	Pretest PSA > 0.5 ng/mL	
Total number of patients		1216	271	945	
Age, yrs		68	66	68	0.0008
		(62, 73)	(61, 71)	(62, 73)	
ISUP grade	1	123	20	103	0.112
	2	356	90	266	
	3	253	62	191	
	4	158	39	119	
	5	245	43	202	
	Unknown	81	17	64	
T stage	2	424	108	316	0.505
	3	567	134	433	
	Unknown	225	29	196	
EAU BCR risk	Low	343	73	270	
	High	752	169	583	
	Unknown	121	29	92	
Initial treatment	RP ± RT	899	202	710	0.66
	RT	304	69	235	
	Unknown	13	1	12	
Time to BCR, months		58	45	60	0.012
		(25, 105)	(18, 95)	(26, 107)	
Time after restaging, months		42	42	42	0.693
		(29, 53)	(27, 53)	(30, 53)	

The *p* values show comparisons between patients with pretest PSA below or above the PSA threshold 0.5 ng/mL. Continuous variables were evaluated with *t*-tests, and categorial variables were evaluated with chi^2^ tests. The Table summarizes continuous characteristics as the median value, with lower and upper quartiles in parentheses. Abbreviations: BCR = biochemical recurrence; EAU = European Association of Urology; ISUP = International Society of Pathology; PSA = prostate specific antigen: RP = radical prostatecomy; RT = radiation therapy; T = tumor.

**Table 2:** Clinical characteristics and restaging PSMA.

Characteristic		Results Number of Sites on Restaging PSMA			*p* Value
		0	1–5	>5	
Number of patients		369	711	123	
Age at diagnosis (years)		67 (62–72)	67 (62–73)	69 (63–74)	0.008
ISUP grades	1	48	60	5	0.26
	2	119	211	26	
	3	74	151	28	
	4	36	103	19	
	5	63	142	40	
	Unknown	29	35	5	
T stage	2	149	243	26	0.001
	3	160	328	72	
Initial treatment	RP	291	515	92	0.26
	RT	78	185	31	
Pretest PSA (ng/mL)		0.595 (0.315, 1.29)	1.89 (0.77, 4.1)	5.455 (2.2, 14)	<0.00005
Follow-up time (months)		43 (31–54)	42 (29–52)	39 (24–50)	0.0034
Number of deaths		19	82	32	<0.0005

Abbreviation: PSMA = prostate specific membrane antigen.

**Table 3:** Pretest PSA and restaging PSMA PET/CT.

Pretest PSA (ng/mL)	Results					
	Restaging PSMA PET/CT					
	Number of Sites			Location of Sites		
	0	1–5	>5	T	N	M
<0.5	152	111	8	33	49	37
>0.5	217	600	115	154	241	320

Abbreviations: T = prostate bed; N = regional lymph nodes; and M = distant organs.

**Table 4:** Initial Cox regression analyses of factors potentially predicting OS.

Clinical Variable	Hazard Ratio	95% CI	*p* Value
Age	0.98	0.94–1.004	0.087
ISUP grade	1.37	1.07–1.76	0.014
T stage	0.68	0.39–1.19	0.18
Initial treatment	1.24	0.63–2.44	0.53
EAU BCR risk groups	0.44	0.15–1.22	0.13
Time to BCR	0.999	0.63–2.44	0.64
Pretest PSA threshold	4.34	1.04–18.2	0.044
PSADT	0.99	0.97–1.006	0.23
PSMA PET/CT site groups	2.59	1.62–4.34	<0.0005

Abbreviations: CI = confidence interval; and PSADT = PSA doubling time.

**Table 5:** Final Cox model for OS.

Variables -> Results			Results
Significant predictive factors			Hazard ratio
Center	0.77	0.67–0.88	<0.0005
ISUP grade	1.25	1.09–1.44	0.001
Pretest PSA threshold	6.26	2.30–17.1	<0.0005
Grouping of no of positive sites on restaging PSMA PET/CT	1.87	1.39–2.51	<0.0005

Abbreviations in Table 5 are like those in Table 4.

## Data Availability

Patient data are not publicly available.
